# Advancing the Genetics of Lewy Body Disorders with Disease‐Modifying Treatments in Mind

**DOI:** 10.1002/ggn2.202200011

**Published:** 2022-08-19

**Authors:** Gilberto Levy, Bruce Levin, Eliasz Engelhardt

**Affiliations:** ^1^ Rio de Janeiro 22441‐012 Brazil; ^2^ Department of Biostatistics Mailman School of Public Health Columbia University New York 10032 USA; ^3^ Instituto de Neurologia Deolindo Couto and Instituto de Psiquiatria Universidade Federal do Rio de Janeiro Rio de Janeiro 22290‐140 Brazil

**Keywords:** dementia with Lewy bodies, disease‐modifying treatments, genetics, Lewy body disorders, Parkinson's disease

## Abstract

In this article, a caveat for advancing the genetics of Lewy body disorders is raised, given the nosological controversy about whether to consider dementia with Lewy bodies (DLB) and Parkinson's disease (PD) as one entity or two separate entities. Using the framework of the sufficient and component causes model of causation, as further developed into an evolution‐based model of causation, it is proposed that a disease of complex etiology is defined as having a relatively high degree of sharing of the component causes (a genetic or environmental factor), that is, a low degree of heterogeneity of the sufficient causes. Based on this definition, only if the sharing of component causes within each of two diseases is similar to their combined sharing can lumping be warranted. However, it is not known whether the separate and combined sharing are similar before conducting the etiologic studies. This means that lumping DLB and PD can be counterproductive as it can decrease the ability to detect component causes despite the potential benefit of conducting studies with larger sample sizes. In turn, this is relevant to the development of disease‐modifying treatments, because non‐overlapping causal genetic factors may result in distinct pathogenetic pathways providing promising targets for interventions.

## Graphical abstract

Lewy body disorders include two of the most common neurodegenerative diseases, dementia with Lewy bodies and Parkinson's disease. While major progress has been made in uncovering the genetic etiology of these diseases, this article argues, for the sake of discovering disease‐modifying treatments, that they should continue to be studied separately rather than combined in genetic studies.

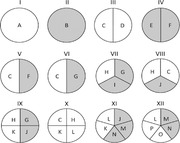

## 1 Introduction

James Parkinson, in 1817, described the condition he called Shaking Palsy or *Paralysis Agitans*,^[^ ^1^ ^]^ which also became known as Parkinson's disease (PD) forty‐five years later when Jean‐Martin Charcot and Alfred Vulpian used the term “maladie de Parkinson” for the first time in 1862.^[^ ^2^ ^]^ Fifty years later, in 1912, Friedrich Heinrich Lewy first described previously unknown neuronal inclusion bodies in the brains of patients with *Paralysis Agitans*,^[^ ^3^ ^]^ which were named after him as “Lewy bodies” just one year later by Gonzalo Rodriguez Lafora.^[^ ^4^ ^]^ In 1923, Lewy^[^ ^5^ ^]^ described clinical and neuropathological aspects of a heterogeneous group of patients with PD, to which he still referred as *Paralysis Agitans*, a certain number of them with dementia (see also Goedert et al.^[^ ^6^ ^]^ and Engelhardt and Gomes^[^ ^7^ ^]^). However, it was only in 1980 that the term “Lewy body disease” was proposed by Kenji Kosaka,^[^ ^8^ ^]^ now understood as a generic term that includes PD, PD dementia (PDD) and dementia with Lewy bodies (DLB), and aligning itself with the notion of a spectrum of Lewy body disorders (LBD).^[^ ^9^, ^10^, ^11^, ^12^ ^]^ In this article, we raise a caveat for advancing the genetics of LBD, given the nosological controversy about whether to consider DLB and PD (with or without dementia) as one entity or two separate entities.

## 2 The Nosological Controversy

The controversy about considering DLB and PDD as one entity or two separate entities (lumping versus splitting) originated from the first DLB Consortium meeting in the mid‐1990s,^[^ ^13^ ^]^ in which “the Consortium members generated en passant the need for a definition of PDD, since it appeared to them that DLB and PDD could not be said to be the same.”^[^ ^12^ ^]^ Because dementia develops in a majority of patients with PD,^[^ ^14^, ^15^ ^]^ being thus properly viewed as an integral part of PD, there is arguably no distinction between lumping DLB with PDD and lumping DLB with PD; we take this perspective throughout this article. Findings from clinical, imaging, neuropathological, and neurochemistry studies comparing DLB and PD^[^ ^9^, ^10^, ^11^, ^12^, ^16^, ^17^, ^18^, ^19^, ^20^, ^21^, ^22^, ^23^, ^24^, ^25^ ^]^ can support either lumping or splitting the two diseases, depending on whether the focus is put on similarities or differences. As an illustration, **Table** **1** shows clinical and pathological findings in favor of lumping versus splitting DLB and PD.

**Table 1:** Clinical and pathological findings in favor of lumping versus splitting DLB and PD^[^ ^10^, ^16^, ^18^, ^20^, ^22^, ^24^ ^]^

	In favor of lumping	In favor of splitting	Comment
Clinical findings	Similar clinical pictures in late stages of the diseases	Different timing of the onset of motor and cognitive manifestations	The choice of one year in the one‐year rule is arbitrary
Pathological findings	Presence of Lewy bodies and Lewy neurites in overlapping distributions and similarly containing alpha‐synuclein aggregates	More severe substantia nigra neuronal loss in PD than in DLB; higher burden of cortical Lewy bodies in temporal and parietal cortex, more frequent and severe alpha‐synuclein load in the CA2 region of the hippocampus, and higher beta‐amyloid load in cortical and subcortical regions in DLB than in PD	Pathological studies typically show the terminal picture of a disease process developing over a period of time, so that they cannot reveal topographic differences in the distribution of Lewy bodies and Lewy neurites during the course of the diseases

DLB: Dementia with Lewy bodies; PD: Parkinson's disease.

Much of the debate about the nosology of LBD has also revolved around the arbitrariness of the one‐year rule, according to which DLB is diagnosed if dementia precedes or occurs within one year of the onset of extrapyramidal motor signs, and PDD is diagnosed if dementia develops one year or more after the onset of extrapyramidal motor signs.^[^ ^13^ ^]^ We have recently proposed a data‐driven alternative to the one‐year rule, based on the examination of the distribution of a timing variable defined as the age of onset of dementia minus the age of onset of extrapyramidal motor signs.^[^ ^26^ ^]^ In 2005, the third DLB Consortium report de‐emphasized the one‐year rule and suggested the following: “Descriptive labels that include consideration of the temporal course [i.e., DLB and PDD] are preferred for clinical, operational definitions”, whereas the “unitary approach to classification may be preferable for molecular and genetic studies and for developing therapeutics.”^[^ ^27^ ^]^ Similarly, in 2007, the DLB/PDD Working Group defended that the distinction between DLB and PDD be made for “routine patient care, clinical interventions and clinical research”, but not “when researching the underlying biology.”^[^ ^10^ ^]^ In 2017, the fourth and latest consensus report of the DLB Consortium moderated this position by stating that the one‐year rule “remains useful, particularly in clinical practice,” while also recommending it for “research studies in which distinction needs to be made between DLB and PDD.”^[^ ^28^ ^]^

## 3 Advancing the Genetics of Lewy Body Disorders

The value of keeping DLB and PD as separate entities for the purpose of clinical practice is hardly debatable, since it is in line with offering patients a specific diagnosis with distinct implications for management decisions and prognosis.^[^ ^12^ ^]^ In turn, the soundness of lumping DLB and PD for research purposes, in particular for studies investigating genetic etiology, deserves careful consideration. When considering the nosology of Mendelian genetic diseases, one faces difficulties presented by two leading principles in genetic nosology, pleiotropism and genetic heterogeneity.^[^ ^29^ ^]^ Pleiotropism refers to different phenotypes resulting from a single etiologic factor or gene (“many from one”), while genetic heterogeneity refers to the opposite situation of a single phenotype resulting from different genes (“one from many”). As stated by McKusick^[^ ^29^ ^]^ in 1969, “Psychologists tell us that we find it easier to recognize similarities than differences. Hence a natural tendency to lumping exists. However, geneticists are forced to be splitters because of their recurrent encounters with genetic heterogeneity in recent years.”

**Figure 1:**
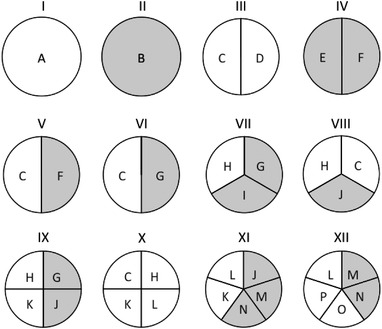
Schematic representation of “causal pies” for a disease of complex etiology. A pie represents a sufficient cause, and a slice represents a component cause, which can be a genetic factor (represented in white) or an environmental factor (represented in gray). A component cause can be shared by two or more sufficient causes, as illustrated by component causes C, F, G, H, J, K, L, M, and N. As exemplified by Parkinson's disease (PD), sufficient cause I represents the more than 20 monogenic causes that have been identified so far; sufficient cause II represents single environmental factors supposedly causing PD independent of genetic factors, such as 1‐methyl‐4‐phenyl‐1,2,3,6‐tetrahydropyridine; and sufficient causes V–IX, XI, and XII represent gene‐environment interactions, which are believed to play a major role in PD. Reproduced with permission.^[^ ^26^ ^]^ Copyright 2020, International Parkinson and Movement Disorder Society, published by John Wiley and Sons.

It is reasonable to expect that one will face more intricate difficulties when dealing with diseases of complex etiology, involving multiple genetic and environmental causal factors. A useful device for thinking about the etiology of complex multifactorial diseases is the sufficient and component causes (SCC) model of causation in epidemiology, which provides a convenient way of conceptualizing biological interactions (i.e., gene‐gene, environment‐environment, and gene‐environment interactions).^[^ ^30^ ^]^ A sufficient cause in the SCC model represents a minimal set of conditions that produce disease, and a given individual may suffer from a disease due to any of many sufficient causes. Each sufficient cause includes one or more component causes (a genetic or an environmental factor), while each component cause can be part of one or more sufficient causes. This can be illustrated graphically with “causal pies”, in which the pie represents a sufficient cause and a slice represents a component cause (**Figure** **1**). Genetic heterogeneity is inherent in the etiology of complex diseases, as evidenced by the notion in the SCC model that a disease can be caused by any of many sufficient causes containing genetic component causes. In this context, pleiotropism can be considered to occur if two diseases have one or more sufficient causes in common, but the operation of pleiotropism can also be considered at the level of the genetic component causes, in which case it is more pervasive.^[^ ^31^ ^]^

### 3.1 A Theoretical Argument

The SCC model has been further developed into an evolution‐based model of causation (EBMC).^[^ ^32^, ^33^ ^]^ Among other things, evolutionary reasoning applied to the SCC model can provide a satisfactory explanation for why common genetic variants have low or medium penetrance, while rare genetic variants have high penetrance. Penetrance generally means the probability that a gene results in a disease or phenotype. Under the SCC model, the penetrance for each genetic component cause can be understood in terms of the number of other necessary component causes participating with it in a sufficient cause. The smaller the number of components in a sufficient cause, the higher the penetrance of each component tends to be (and vice versa), because the probability that any genetic component will be phenotypically expressed increases as the number of necessary conditions decreases. This is consistent with the notion put forward by Lander and Schork^[^ ^34^ ^]^ that the penetrance function, given by the probability of disease for each genotype, may depend on other genes and environmental factors. Then evolutionary reasoning implies that, since phenotypic expression is necessary for the action of natural selection, low‐ or medium‐penetrance variants would become common because, in the absence of one or more of their several causal partners, meaning the other component causes in a sufficient cause, the action of natural selection does not occur. In other words, it is as if they were shielded from the action of natural selection by the absence of their causal partners. High‐penetrance variants, or at the extreme fully penetrant monogenic causes of disease, are rare because they need few or no causal partners to produce phenotypic expression, such that they are more consistently subjected to natural selection.

There is evidence that both common and rare genetic variants play a role in the causation of complex diseases (i.e., a combination of the common disease‐common variant and the common disease‐rare variant hypotheses),^[^ ^35^ ^]^ as has been specifically advanced for the sporadic forms of PD^[^ ^36^, ^37^ ^]^ and DLB.^[^ ^38^, ^39^, ^40^ ^]^ Further, the occurrence of Mendelian (familial) forms of neurodegenerative diseases, most prominently PD and Alzheimer's disease (AD), has motivated the view that there exists a “genetic dichotomy” for these diseases.^[^ ^41^, ^42^ ^]^ While the consideration of a genetic dichotomy is certainly useful for clinical purposes (e.g., other than genetic counseling considerations, Mendelian forms of PD can have characteristic manifestations in addition to parkinsonism), the distinction is not clear‐cut at a fundamental level. Blauwendraat et al.^[^ ^37^ ^]^ noted in a recent review of PD genetics that the disease does not manifest in some carriers of supposed monogenic causes (incomplete penetrance), suggesting that “additional genetic or environmental factors affect the disease process in addition to the single variant of interest.” They also noted that the characterization of most cases of PD as sporadic (or idiopathic) is an oversimplification, because we now know that “The implication that typical PD arises spontaneously or with an unknown cause or origin is inaccurate.” This somewhat blurred distinction supports the joint consideration of Mendelian and sporadic forms of PD. In the EBMC, rather than considering Mendelian forms of complex diseases separately, the monogenic causes and the sufficient causes involving a few or several variants are seen as part of a continuum according to the number of component causes in each sufficient cause (Figure 1).^[^ ^32^ ^]^

With respect to malignant neoplastic diseases, Galvan et al.^[^ ^43^ ^]^ considered that “Regardless of frequency (i.e., common, rare, very rare) and type (i.e., SNP [single‐nucleotide polymorphism] or CNV [copy‐number variants]), it is likely that hundreds or even thousands of genetic variants are implicated in cancer risk.” This genetic architecture, together with the contribution of environmental factors, is consistent with the operation of at least hundreds of sufficient causes in different types of cancer, and the same is true for most if not all complex diseases.^[^ ^32^ ^]^ Among neurodegenerative diseases, PD provides a good example. In 2014, 28 genetic risk variants for PD had been identified through genome‐wide association studies.^[^ ^44^ ^]^ Yet in a 2016 review of PD genetics, Hernandez et al.^[^ ^36^ ^]^ considered that “Since identified PD mutations [18 with Mendelian inheritance at the time] and risk variants explain only a small percentage of disease burden, additional genetic determinants of PD remain to be discovered.” In 2019, the number of causative genes surpassed 20 and the number of identified risk loci rose to 90.^[^ ^37^, ^45^ ^]^ However, Blauwendraat et al.^[^ ^37^ ^]^ argued that, based on the estimated heritable component of PD due to common genetic variability, “many more risk variants are yet to be discovered.” As for environmental factors, about 10 metals and pesticides have been associated with PD.^[^ ^46^ ^]^ These environmental factors can combine with genetic risk variants to produce sufficient causes involving gene‐environment interactions, which are believed to play a major role in the etiology of PD.^[^ ^47^, ^48^, ^49^ ^]^

If for Mendelian diseases, as put by McKusick,^[^ ^29^ ^]^ “Few would quibble with the statement that the phenotype resulting primarily from a specific and unitary factor is an entity,” for multifactorial diseases one can only go so far as to say that many would agree about the role of etiology in defining an entity. Most importantly, exactly how etiology is to be taken into account in defining a multifactorial entity is not clear. On one hand, Aarsland et al.^[^ ^11^ ^]^ argued that the occurrence of DLB and PD phenotypes in different members of the same family supported lumping the two diseases, based on a review of familial occurrence of dementia and parkinsonism that found 12 out of 24 families in which members presented as either DLB or PDD^[^ ^50^ ^]^ and the report of a Belgian family also with both clinical presentations.^[^ ^51^ ^]^ More recently, Quadri et al.^[^ ^52^ ^]^ reported additional European families with dominantly‐inherited disease presenting as DLB or PD. As indicated above, Mendelian forms of complex diseases can be represented by a sufficient cause containing only one genetic component cause under the framework of the EBMC. This would account, for example, for the relatively small proportion of PD cases due to monogenic causes.^[^ ^37^, ^49^ ^]^ Likewise, PD cases caused by exposure to a single environmental factor, such as 1‐methyl‐4‐phenyl‐1,2,3,6‐tetrahydropyridine,^[^ ^53^ ^]^ can be represented by a sufficient cause containing only one environmental component cause (see Figure 1). The fact that DLB and PD phenotypes co‐occur within a number of families suggests that there may be a corresponding number of sufficient causes in common between the sets of sufficient causes for the two diseases, which is not a strong reason for lumping given the operation of at least hundreds of sufficient causes.

On the other hand, it can be argued that setting the boundaries between DLB and PD correctly, in the first place, is what will allow getting to more fully know their causes.^[^ ^54^ ^]^ This is to say that we should consider how to define disease in the setting of complex etiology with a view to facilitating discoveries. Under the EBMC framework, we have proposed that a disease of complex etiology is defined as having a relatively high degree of sharing of the component causes (i.e., a relatively low degree of heterogeneity of the sufficient causes) among those affected by the disease in the population, such that the overall frequencies of component causes in a sample of patients with the disease would tend to be higher than in a sample that also included patients with another disease.^[^ ^26^ ^]^ For a single component cause, one can of course observe an increased or decreased frequency by lumping one disease with another with a higher or lower frequency. For example, comparing combined DLB/PDD with PDD alone, two genetic association studies reported a higher frequency of the *APOE* ε4 allele (25.9% in DLB/PDD versus 19.1% in PDD alone)^[^ ^55^ ^]^ and a lower frequency of genotype AA in the *TFAM* gene (38.5% vs. 44.4%).^[^ ^56^ ^]^ The notion of sharing of component causes in our definition refers to overall frequencies of component causes in an average sense; for a given disease, we might in principle calculate for each component cause the proportion of all sufficient causes in the population in which it participates, and then average the proportions over all component causes.

Based on a definition of disease taking into account “common etiology, pathophysiology, clinical presentation, and course”, Aarsland et al.^[^ ^17^ ^]^ expressed an inclination toward lumping DLB and PDD in these terms: “The available evidence suggests that the heterogeneity within the two syndromes may be greater than the differences between them.” Based on our definition, this sort of reasoning pertains more narrowly to the level of etiology: If the sharing of component causes within each of two diseases is higher than their combined sharing (i.e., greater heterogeneity between than within them), lumping is not warranted; and if the sharing of component causes within each of two diseases is similar to their combined sharing, lumping is warranted. Importantly, the definition has the property of increasing the chances of discovering the causes of complex diseases, by maximizing the overall frequencies of component causes in samples of etiologic studies. In the first scenario above, were we to lump DLB and PD (not warranted under the definition), we would tend to decrease the overall frequencies of component causes in study samples; in the second scenario, were we to lump DLB and PD (warranted under the definition), we would not substantially affect the overall frequencies of component causes. The problem is that we don't know whether the separate and the combined sharing are similar before we conduct the etiologic studies. Therefore, this can be seen as a cautionary note against lumping diseases of complex etiology, since it can decrease the ability to detect component causes despite the potential benefit of conducting studies with larger sample sizes.

### 3.2 The Empirical Evidence

**Table** **2** summarizes genetic studies on DLB relevant to the investigation of widespread genetic sharing between DLB and PD. So far, empirical studies do not support the possibility of widespread sharing of genetic factors between the two diseases. For instance, a study that assessed mutations in established causal genes for PD and AD among 99 patients with DLB found evidence of a contribution of these genetic factors only to a small percentage of subjects.^[^ ^57^ ^]^ In another association study of a much larger cohort of DLB cases and controls assessing 54 genomic regions previously implicated in PD or AD, Bras et al.^[^ ^58^ ^]^ confirmed the association of the *APOE* locus with DLB and found that the *SNCA* and *SCARB2* loci are also associated with DLB, although with a different profile than the associations reported in PD. Performing a whole exome sequencing of a cohort of 1118 patients with DLB, focused on 60 causal genes of other neurodegenerative diseases (including AD, PD, and frontotemporal dementia, among others), Orme et al.^[^ ^59^ ^]^ found rare missense variants of unknown significance. In a genome‐wide analysis quantifying the sharing of genetic risk among DLB, PD, and AD, Guerreiro et al.^[^ ^60^ ^]^ reported estimates of genetic correlation of 0.578 for DLB and AD (0.332 excluding the *APOE* locus) and 0.362 for DLB and PD. That is, according to this analysis DLB shares more pleiotropic genetic determinants with AD than with PD. However, it is important to note that genetic correlation can artifactually arise due to a proportion of cases of one disease being misclassified as another.^[^ ^31^ ^]^

**Table 2:** Genetic studies on DLB relevant to the investigation of widespread genetic sharing between DLB and PD

Authors	Sample and diagnosis	Country/continent or ethnicity	Type of study and candidate genes/regions tested	Main study findings
Meeus et al.^[^ ^57^ ^]^	99 patients with DLB 75 patients with PDD 626 controls	Belgium	Gene‐based mutation analysis of established causal genes for PD (*SNCA*, *LRRK2*, *PARK2*, *PINK1*, *DJ‐1*, *GBA*) and AD (*APP*, *PSEN1*, *PSEN2*, *MAPT*, *PGRN*, *TARDBP*)	Among PD genes, one *SNCA* duplication, the *LRRK2* R1441C founder mutation, and four novel heterozygous missense variants with unknown pathogenicity were detected; among AD genes, proven pathogenic missense mutations in *PSEN1* and *PSEN2* and two novel missense variants in *PSEN2* and *MAPT* were detected
Bras et al.^[^ ^58^ ^]^	788 patients with DLB (667 pathologically confirmed) 2624 controls	Europe and North America	Association study of 54 genomic regions previously implicated in PD or AD	*APOE* associated with DLB; *SNCA* and *SCARB2* loci also associated with DLB, although with a different profile than the associations in PD
Guerreiro et al.^[^ ^60^ ^]^	788 patients with DLB (667 pathologically confirmed) 804 patients with PD 959 patients with AD (113 pathologically confirmed) 2806 controls	Europe and North America	Genome‐wide genetic correlation analysis	Genetic correlation of 0.578 for DLB and AD (0.332 excluding the *APOE* locus) and 0.362 for DLB and PD
Guerreiro et al.^[^ ^61^ ^]^	1743 patients with DLB (1324 pathologically confirmed) 4454 controls	Europe, North America, and Australia	Genome‐wide association study	Confirmed previously reported associations of *APOE*, *SNCA*, and *GBA* with DLB; some evidence for a novel candidate locus (*CNTN1*)
Orme et al.^[^ ^59^ ^]^	1118 patients with DLB (all pathologically confirmed) 432 controls	Caucasian	Whole exome sequencing focused on 60 genes causative of monogenic neurodegenerative diseases (*APP*, *ATP1A3*, *CCNF*, *CHCHD10*, *CHCHD2*, *CHMP2B*, *COL4A1*, *CSF1R*, *CYLD*, *DCTN1*, *DNAJC13*, *DNMT1*, *FUS*, *GCH1*, *GRN*, *HNRNPA1*, *HNRNPA2B1*, *ITM2B*, *LRRK2*, *MAPT*, *MATR3*, *NOTCH3*, *PRKAR1B*, *PRNP*, *PSEN1*, *PSEN2*, *SERPINI1*, *SNCA*, *SNCB*, *SQSTM1*, *TARDBP*, *TBK1*, *TIA1*, *TMEM230*, *TUBA4A*, *VCP*, *VPS35*, *ATP13A2*, *DNAJC6*, *FBXO7*, *HTRA1*, *OPTN*, *PANK2*, *PARK2*, *PARK7*, *PINK1*, *PLA2G6*, *SPG11*, *SYNJ1*, *TH*, *TREM2*, *TYROBP*, *VPS13C*, *POLG*, *ATP6AP2*, *RAB39B*, *UBQLN2*, *GBA*, *APOE*, *PLCG2*)	Rare missense variants of unknown significance were found in *APP*, *CHCHD2*, *DCTN1*, *GRN*, *MAPT*, *NOTCH3*, *SQSTM1*, *TBK1*, and *TIA1*; a pathogenic *GRN* p.Arg493* mutation was identified
Chia et al.^[^ ^62^ ^]^	2591 patients with DLB (1789 pathologically confirmed) 4027 controls	European ancestry	Genome‐wide association study	Genome‐wide association analysis identified five significant loci (*GBA*, *BIN1*, *TMEM175*, *SNCA‐AS1*, and *APOE*); gene‐level aggregation testing identified *GBA* as a pleomorphic risk gene; strong cis‐eQTL colocalization signals at the *TMEM175* and *SNCA‐AS1* loci were detected

AD, Alzheimer's disease; DLB, Dementia with Lewy bodies; PD, Parkinson's disease; PDD, Parkinson's disease dementia.

In the first genome‐wide association study in DLB, including 1743 patients and 4454 controls, Guerreiro et al.^[^ ^61^ ^]^ confirmed previously reported associations of *APOE*, *SNCA*, and *GBA* in candidate gene studies. Although one of these risk loci is shared with AD (*APOE*) and two are shared with PD (*SNCA* and *GBA*), the most significantly associated SNP at the *SNCA* locus for PD was not significant for DLB (the association at the *SNCA* locus for DLB was mediated by a SNP at a different location), and a systematic assessment of other genetic loci previously associated with AD or PD showed no evidence of significant associations with DLB. Most recently, in a second genome‐wide association study in DLB including data from 2591 patients and 4027 controls, Chia et al.^[^ ^62^ ^]^ identified five significant loci: three previously identified (*APOE*, *SNCA*, and *GBA*) and two new loci, one of which had been previously implicated in AD (*BIN1*)^[^ ^63^ ^]^ and the other in PD (*TMEM175*).^[^ ^64^ ^]^ Again, the authors reported “a notably different profile at the *SNCA* locus” in DLB and PD, suggesting that “the regulation of *SNCA* expression may be different” in the two diseases. This study also tested associations of AD and PD genetic risk scores, derived from meta‐analyses of genome‐wide association studies, with DLB disease status. The associations with DLB status were significant for both AD and PD genetic risk scores, but they were stronger for AD scores, even after adjustment for *APOE*.^[^ ^62^ ^]^

## 4 Conclusion

In connection with the practical character of medicine, nosological classifications ought to take into account their ability to bring about benefits for patients. Friedman^[^ ^23^ ^]^ noted the advantages of considering DLB and PDD as the same entity, such as resources for PD research and support also being used for DLB, and medications approved for dementia and psychosis in PD being extended to patients with DLB. However, by allowing to more fully know the genetic etiology of LBD, keeping DLB and PD as separate entities favors the ultimate goal of discovering disease‐modifying treatments, because non‐overlapping causal genetic factors may result in distinct pathogenetic pathways providing promising targets for interventions. This is to say that, given that DLB and PD may not share most of their genetic etiologic factors as well as pathogenetic pathways, it is not wise to infer from their clinical and pathologic commonalities that they will respond to the same disease‐modifying treatments. As pragmatically stated by Revuelta and Lippa^[^ ^20^ ^]^ in 2009, “DLB and PDD are one entity when therapies are developed that influence pathological alpha‐synuclein protein aggregation and other common biological alterations.”

Counter to earlier consensus suggestions that DLB and PDD might be lumped for basic biology and treatment studies, the Lewy Body Dementia Association Scientific Advisory Council stated this in 2016: “Both phenotypes should be included in clinical trials of new therapies for Lewy body dementia (as opposed to lumping them into one phenotype) until more research improves knowledge about the differences and similarities between PDD and DLB.”^[^ ^65^ ^]^ Consistent with the theoretical argument presented here, we have suggested that studies on the etiology and pathogenesis of LBD should continue to analyze DLB and PD separately, preferably by replacing the arbitrary one‐year rule with the proposed data‐driven alternative.^[^ ^26^ ^]^ If the basic biology findings support lumping, the investigation of a common disease‐modifying treatment for LBD may be reasonably conducted. Otherwise, if the basic biology findings support splitting, further research may eventually replace clinical rules with biomarkers for differentiating DLB and PD, which may then be used to investigate disease‐modifying treatments in the two diseases separately.

## Conflict of Interest

The authors declare no conflict of interest.

## Peer Review

The peer review history for this article is available in the Supporting Information for this article.

## Supporting information




**Supplementary Information**: Record of Transparent Peer Review
